# Forearm Posture and Mobility in Quadrupedal Dinosaurs

**DOI:** 10.1371/journal.pone.0074842

**Published:** 2013-09-18

**Authors:** Collin S. VanBuren, Matthew Bonnan

**Affiliations:** 1 Department of Ecology and Evolutionary Biology, University of Toronto, Toronto, Ontario, Canada; 2 Biology Program, The Richard Stockton College of New Jersey, Galloway, New Jersey, United States of America; University of Pennsylvania, United States of America

## Abstract

Quadrupedality evolved four independent times in dinosaurs; however, the constraints associated with these transitions in limb anatomy and function remain poorly understood, in particular the evolution of forearm posture and rotational ability (i.e., active pronation and supination). Results of previous qualitative studies are inconsistent, likely due to an inability to quantitatively assess the likelihood of their conclusions. We attempt to quantify antebrachial posture and mobility using the radius bone because its morphology is distinct between extant sprawled taxa with a limited active pronation ability and parasagittal taxa that have an enhanced ability to actively pronate the manus. We used a sliding semi-landmark, outline-based geometric morphometric approach of the proximal radial head and a measurement of the angle of curvature of the radius in a sample of 189 mammals, 49 dinosaurs, 35 squamates, 16 birds, and 5 crocodilians. Our results of radial head morphology showed that quadrupedal ceratopsians, bipedal non-hadrosaurid ornithopods, and theropods had limited pronation/supination ability, and sauropodomorphs have unique radial head morphology that likely allowed limited rotational ability. However, the curvature of the radius showed that no dinosaurian clade had the ability to cross the radius about the ulna, suggesting parallel antebrachial elements for all quadrupedal dinosaurs. We conclude that the bipedal origins of all quadrupedal dinosaur clades could have allowed for greater disparity in forelimb posture than previously appreciated, and future studies on dinosaur posture should not limit their classifications to the overly simplistic extant dichotomy.

## Introduction

Posture and locomotion are two important, interdependent factors affecting many biological aspects of tetrapods (e.g., [1]–[3]). Transition in posture and locomotion reflect parallel changes in ecology and may allow for the expansion of a clade into previously unoccupied niches (e.g., [4]–[6]). Such transitions in the theropod dinosaur lineage that led to modern birds (e.g., [7], [8]) and in human evolution (e.g., [9]–[11]) have been well studied, although many questions still remain. The rare postural transitions from bipedality to quadrupedality in some dinosaurs has recently been shown to have evolved through highly disparate pathways [12]–[14]. Therefore, constraining postural and locomotor abilities in these groups is critical for determining biomechanical or ecological selective pressures acting on these groups.

Dinosaurs independently evolved quadrupedality secondarily at least four times in their evolutionary history in the long-necked sauropodomorphs, horned and frilled ceratopsians, duck-billed ornithopods, and the armored thyreophorans, making them an ideal clade in which to study evolutionary trends related to forelimb posture. The transition from bipedality to quadrupedality is a rare transition in vertebrate evolution, occurring only once outside Dinosauria in silesaurid dinosauriforms [15]. Previous research on forelimb posture in dinosaurs has largely focused on ceratopsids [16]–[23] and sauropodomorphs [24]–[30]. In ceratopsids, it has been recognized that forelimb posture may have played a role during intraspecific combative behavior (e.g., [31]). Sauropodomorphs are a model clade for understanding the relationship between increasing body mass (gigantism) and forelimb anatomy [1], [2], [32]. Studies on postural evolution using transitional forms such as *Aardonyx* [28], ontogenetic changes in *Massospondylus* [29], and morphometric studies [26], [30] have given great insight into the anatomy and evolution of forelimb posture in sauropodomorphs. However, few locomotor-based functional studies on ceratopsians include non-ceratopsid neoceratopsians [16], [33] or any other marginocephalian and are largely qualitative [17]–[19], [21]. While qualitative studies are important for our understanding of forelimb posture in ceratopsids and other quadrupedal dinosaurs, they are not repeatable and many produce conflicting results due to uncertainties in the articulations of the pectoral girdle and forelimb elements [34]–[37]. Furthermore, recent studies indicate that archosaurs have a significant amount of cartilage covering the epiphyses of their long bones [34]–[36], thereby limiting our confidence in assigning forelimb posture based on qualitative interpretations of long bone articulation in extinct archosaurs.

Studies on dinosaur posture and locomotion categorize taxa using a dichotomy of sprawling or parasagittal forelimb posture (e.g., [17]–[19]). In a sprawling posture, the humerus is directed laterally, and the radius and ulna lie parallel to one another. In a parasagittal posture, forelimb is brought under the body, and the radius crosses the ulna so that the manus can remain directed cranially. While such a strict separation is not reflective of true postural disparity of vertebrates, some general patterns of associated locomotion and biomechanics are correlated with each postural type. As terrestrial vertebrates increase in body mass, their limbs must become increasingly more columnar to maintain appropriate safety factors during locomotion [1], [2]. This scaling relationship suggests that dinosaurs of large body mass would require a parasagittal or semi-sprawled gait. Locomotion is also affected by posture, particularly in the axial column. Animals with sprawling limbs utilize undulations of the vertebral column to aid with locomotion, whereas animals with parasagittal postures rely on flexion and extension of the limbs to propel the body forward (e.g., [38]). Therefore, by understanding aspects of limb posture, reconstructions of kinematic locomotion can be better constrained.

**Figure 1:**
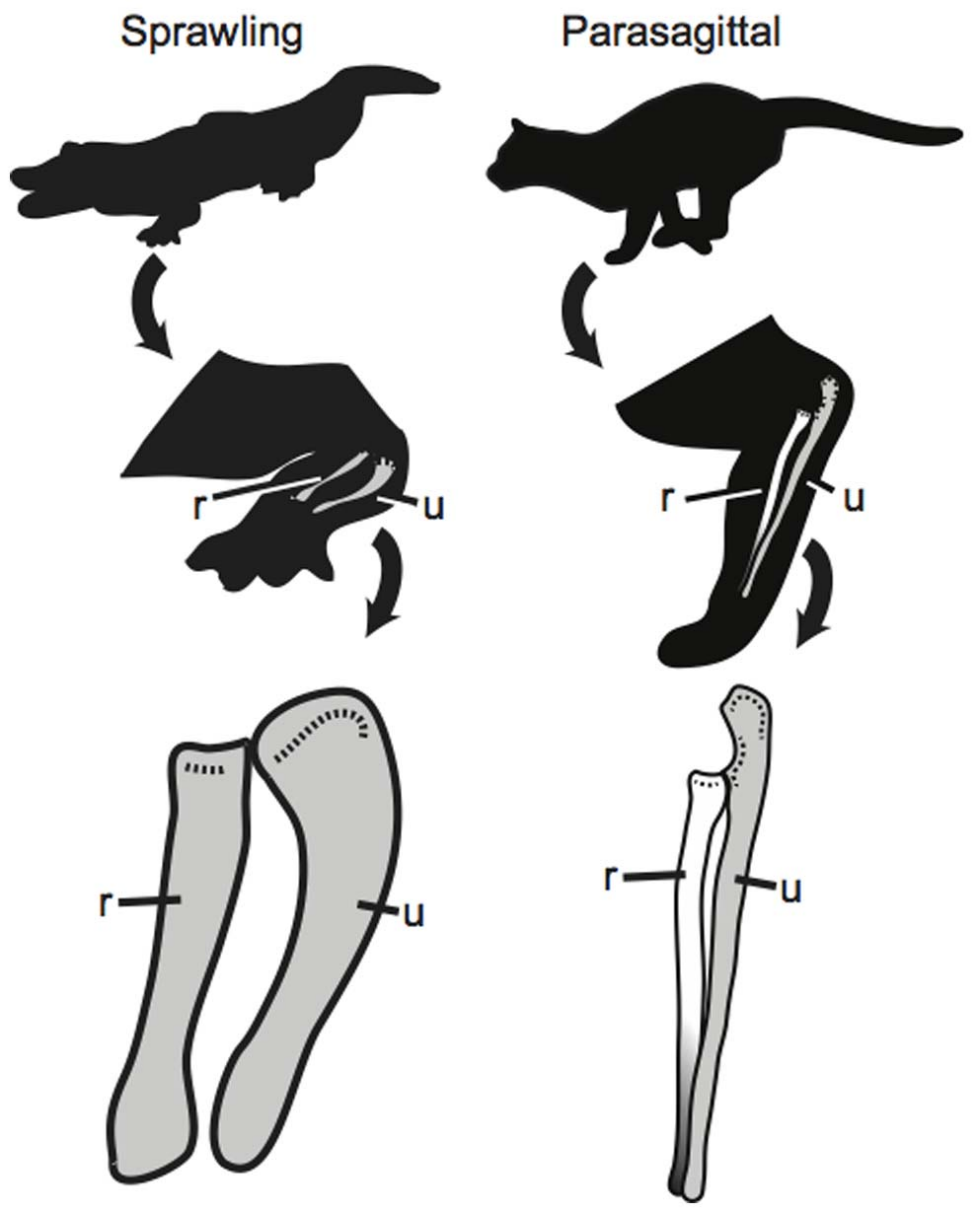
Antebrachia of sprawling and parasagittal taxa. The parallel radius of a sprawling alligator compared to a radius that crosses the ulna in a parasagittal cat. **r** = radius; **u** = ulna.

**Figure 2:**
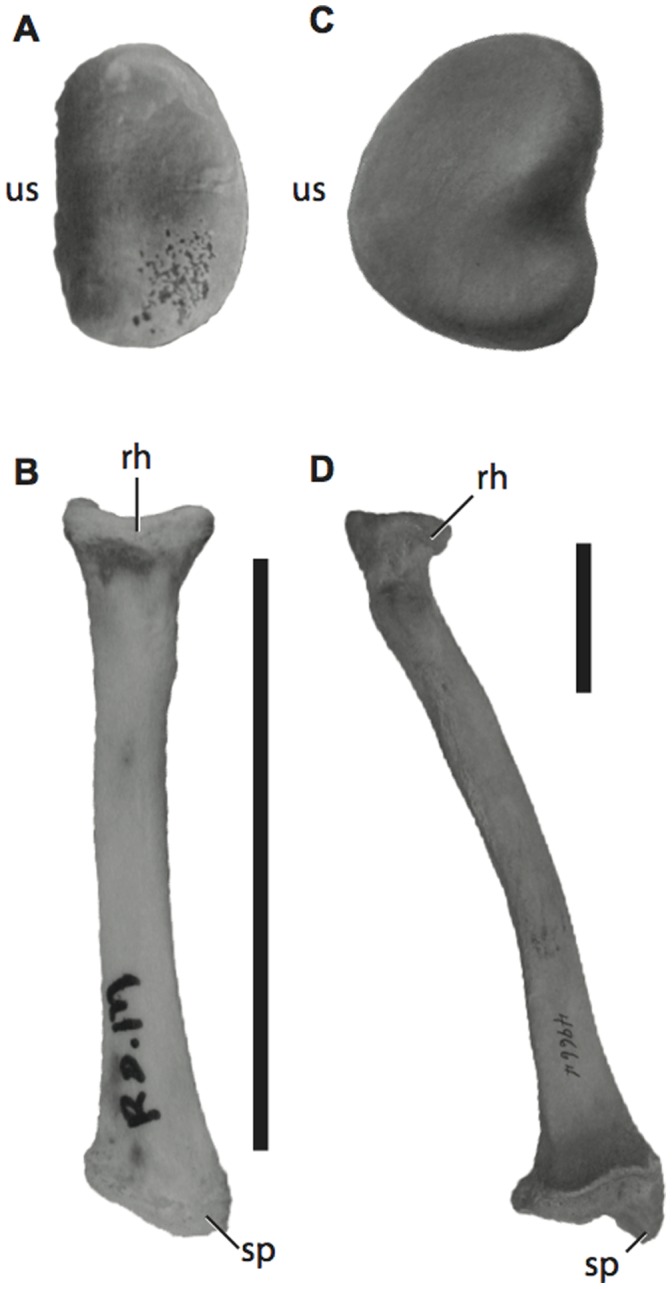
Radii of sprawling and parasagittal extant taxa. The radial head (A) and long axis (B) of *Caiman crocodylus* (ROM R7719) shows the flattened ulnar articular surface and relatively straight long axis typical of sprawling taxa. The radial head (C) and long axis (D) of *Ursus americanas* (USNM 49664) shows the rounded ulnar articular surface and curved long axis typical of parasagittal mammals and chameleons. Scale bar = 5 cm. Radial heads not to scale. **rh**, radial head; **sp**, styloid process; **us**, ulnar articular surface.

The radius is an important element of the forelimb for quadrupedal locomotion, but it is often neglected in studies on dinosaur locomotion (but see [12], [39]). In sprawling taxa, or those with a humerus projected laterally from the midline, the radius lies parallel and medial to the ulna (Fig. 1) [40] and is straight (Fig. 2A, B). In many parasagittal animals, the radius is lateral to the ulna proximally, but it curves about the ulna so that distally it is medial to the ulna [41] (Fig. 1; Fig. 2C, D). A crossed radius is essential to obtain a parasagittal forelimb posture for many extant mammals because it allows the manus to remain directed cranially (be pronated) when the forelimb is drawn near the midline of the body. Alternatively, the manus would direct laterally as the forelimb was drawn near the midline of the body if the radius was unable to cross the ulna. Arboreal chameleons have converged upon a similar radial morphology as they have adopted semi-parasagittal forelimb postures at certain points in their stride [3], [42]. The radius is also known to play an important role in pronation (palmar surface of the manus ventral) and supination (palmar surface of the manus dorsal) abilities in extant mammals because the shape of the radial head and the curvature of the radial diaphysis affect the ability of the radius to rotate about the ulna while maintaining its articulation with the distal humerus and proximal ulna [39], [43], [44]. This freedom to actively manipulate forelimb elements has many ecological benefits in mammals and arboreal chameleons (e.g., [3], [45]–[47]). In contrast, limiting this mobility may have been essential for the acquisition of quadrupedality in dinosaurian taxa [28], suggesting that obligate quadrupeds with limited pronation ability may serve as analogues for understanding dinosaur locomotion. While many studies on forelimb posture in extinct taxa have focused on articulations between the scapula, humerus, ulna, and manus, or use muscle scars to reconstruct posture and range of motion (e.g., [17]–[19], [48]), few have attempted to quantify the morphology of forelimb bones (but see [12]) and of these, none have attempted to correlate morphology with a specific posture or rotational ability within the context of a disparate extant data set.

The goal of this study is to quantify the curvature of the radius and the morphology of the radial head to quantitatively predict forearm posture and mobility in dinosaurs by first assessing if these metrics accurately predict posture and rotational ability in extant taxa. Under the assumption that the aspects of radial morphology listed above affect forearm posture and range of motion, we predict that dinosaur radii should more closely resemble those of mammals if they had more parasagittal forelimbs capable of active pronation/supination or those of squamates and crocodylians if their forelimbs were sprawled with limited mobility.

## Materials and Methods

### Materials

A previous study by MacLeod and Rose [39] used the morphology of the radial head to examine locomotor patterns in Paleogene mammals using extant analogs and worked under the hypothesis that radial head morphology is correlated with the degree of active pronation and supination ability. We are utilizing MacLeod and Rose’s [39] methods to examine forelimb posture and rotational ability in dinosaurs. Theoretically, mammalian-style pronation ability, in which the distal radius rotates about the long axis of the ulna to pronate or supinate the manus, and parasagittal posture can only be achieved with a radius with a rounded radial head and a curved diaphysis, and we use these assumptions as our criteria for determining forearm posture and pronation ability. Extant ungulates (e.g., *Equus*) do not cross the radius over the ulna, yet still have an upright, parasagittal forelimb posture. However, the radius in these taxa is greatly enlarged, and the ulna is significantly reduced. This morphology is not seen in dinosaurs, nor is the converse (enlarged ulna, reduced radius). We therefore chose extant taxa that possess a load-bearing radius that is roughly equal in size with the ulna. Varanid lizards are also capable of pronation and supination but in a way dissimilar to that of mammals [49]. The curvature of the radius in mammals should allow the radial head to remain in the same plane while pronating or supinating. In varanids, the radial head becomes slightly displaced and the distal radius must diverge from the distal ulna when the manus is supinated [49]. Arboreal chameleons can obtain parasagittal forelimb postures [3], [42], but it is unclear how their active pronation ability compares to that of mammals or varanids. Because a mammalian comparison is often invoked for dinosaur forelimb reconstructions (e.g., [17]), our analyses are specifically meant to test for mammalian-style pronation and supination ability only.

Data were collected from 293 specimens from paleontological and extant osteological collections at the American Museum of Natural History (AMNH), Carnegie Museum of Natural History (CM), Field Museum of Natural History (FMNH), Royal Ontario Museum (ROM), Royal Tyrrell Museum (RTMP), United States National Museum (USNM), and Western Illinois University (WIU). The data set (Table S1; File S1) includes 189 mammals, 49 non-avian dinosaurs, 35 squamates, 16 birds, and 5 crocodilians. Only one specimen per species was used for extant taxa as to not overinflate the effect each taxon may have on the analyses. However, because the morphology of fossils can be altered during taphonomic processes, we chose to include multiple members of the same species for extinct taxa when available and only used specimens that did not appear to have significant taphonomic distortion. Photographs of the radial head in proximal view and long axis in ulnar view of most radii were taken using a Sony Cybershot 4.1 megapixel camera on a level tripod, but photographs of the bird taxa and four dinosaurs were taken with a Canon 10.1 megapixel DSLR camera. For consistency in our morphometric analyses, photographs were taken at an orthogonal position from the camera lens. Linear measurements of the proximal-distal straight length, length of the arc of curvature (measured from the proximal epiphysis to the distal epiphysis along the curved diaphysis), and length to the bicipital tubercle (from the proximal epiphysis to the insertion of m. biceps brachii) were also taken, but the amount of missing data and major incongruences between the insertion of m. biceps brachii in mammals, squamates, and archosaurs (e.g., [50], [51]) limited their usefulness for our study.

To examine differences among extant taxa only, we grouped these taxa into Monotremata (*n = *2), Marsupalia (*n* = 12), terrestrial Eutheria (*n* = 106), Squamata (*n = *35), Crocodylia (*n* = 5), Cetacea (*n = *39), Pinnipedia and Sirenia (*n* = 30), and Aves (*n* = 16) (Table S1, ‘Extant Analyses Value’). In our analyses of radial morphology in extinct taxa, extant terrestrial taxa were grouped as either ‘sprawled, limited pronators’ (crocodylians, non-chalaeleonid squamates, and monotremes, *n = *36) or ‘parasagittal, active pronators’ (therian mammals and chameleons, *n* = 121), and dinosaurs were grouped by higher clades [Ceratopsia (*n* = 8), Ornithopoda (*n* = 16), Thyreophora (*n* = 6), Sauropodomorpha (*n* = 10), and Theropoda (*n* = 9); (Table S1, ‘Terrestrial Analyses Values 1′)] to better capture inter-clade differences [12]. Eutherians and marsupials were found to be significantly different in their radial head morphology (see below), so these two groups were separately compared to dinosaurian groups to determine any effect of this difference on the results. Chameleons were also separated from other squamates because they have adopted a unique lifestyle among squamates [3], [42], and it is currently unclear how chameleons differ in pronation/supination from the described mammalian- and varanid-styles. Because a debate over facultative or obligate bipedality for hadrosaurids exists (e.g., [12], [14], [52]–[55]) and our data set contains ornithopod taxa generally accepted to be bipedal (e.g., *Parksosaurus*), we ran our analyses with ornithopods grouped split into hadrosaurids (*n = *12) and non-hadrosaurid (*n* = 4) ornithopods to test for more specific intertaxon differences (Table S1, ‘Terrestrial Analyses Value 2′). The sprawled and parasagittal classifications are likely to capture both postural and antebrachial rotational differences between the groups, although active pronation in some groups of eutherian mammals is much greater (e.g., primates) than other groups (e.g., canids). However, without a more precise understanding of the differences in active pronation ability within these broad taxonomic groups, we used a generalized approach as a baseline upon which more detailed future experiments may be built.

### Angle of Curvature

**Figure 3:**
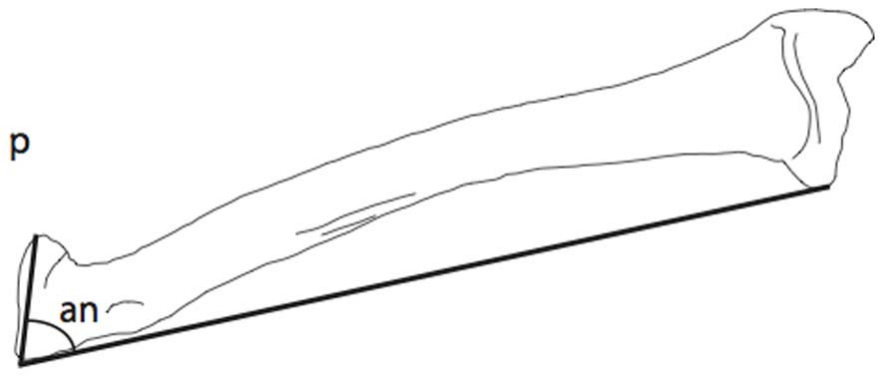
Angle of curvature measurement. The angle of curvature was calculated using ImageJ. The vectors for the angle are from the distal medial end up to the radial head and across the radial head. **an**, angle measurement; **p**, proximal.

Mammalian radii have a curved diaphysis that allows the radius to cross the ulna. This curvature is necessary for both mammalian-style active pronation and for directing the manus cranially when the forelimb is parasagittal. We predicted that the degree to which the radius curves about the ulna should separate sprawling and parasagittal taxa based on the orientation of the radius in the two groups. Our metric for the angle of curvature represents the relationship between the plane of the radial head and the plane of the long axis of the radius. An angle of curvature of 90 degrees should represent a straight radius unable to cross the ulna, and an angle of greater than or less than 90 degrees should indicate a curved radius able to cross the ulna [56]. The angle of curvature was measured using ImageJ [57] using three points at the lateral distal end of the radius, the proximal, lateral radial head, and across the radial head to the medial surface (Fig. 3). Because the angle of curvature was non-normally distributed, we used a multiple comparisons Kruskal-Wallis test with a Bonnferroni-corrected *p*-value using the R package pgirmess [58].

### Geometric Morphometrics

The radial head interacts with the ulna and distal humerus proximally and is the rotation point for active pronation and supination in mammals [39]. We chose to use geometric morphometric techniques for their utility in quantifying morphological disparity by simplifying complex shapes using landmarks and semi-landmarks. An outline analysis of the radial head was most appropriate because the radial head lacks easily identifiable landmarks that are consistent among all taxa examined. Sliding semi-landmarks reduce the amount of variance and do not assume homology like normal landmarks. Photographs were first digitized using TPSUTIL [59]. Outlines of the radial head were created using the left radius or by mirroring the photograph of the right radius in TPSDIG2 [59] (File S1). Specimens were traced from the middle of the ulnar articular surface (the location of the first semi-landmark) clockwise, and the number of semi-landmarks was then adjusted to 20 for each specimen. A landmark was placed at the middle of the ulnar articular surface because it was the most definitive landmark common to all taxa. Curves (semilandmarks) were converted to landmarks using TPSUTIL [59] and then all 21 points (previously 20 semilandmarks and one landmark) were converted to a single curve using CoordGenMac7a [60]. Twenty-one points is an arbitrary number that was easy to use in our statistical analyses and created an appropriate number of variables given our sample size. Although the number of semi-landmarks is important when comparing similar shapes (e.g., intraspecific studies), the amount of variation in interspecific studies allows for more freedom when choosing the number of sliding semi-landmarks [61]. Our arbitrary number should therefore not significantly affect our results.

Geometric morphometric approaches remove the influence of isometric size, location, and orientation of landmark configurations through a generalized Procrustes analysis in partial Procrustes superimposition in relation to the consensus (a mean shape with the lowest sum of squared Procrustes distances from all landmark configurations) [62]–[66]. When using sliding semi-landmarks, a step is added to the generalized Procrustes analysis to reduce variation tangential to the curve by sliding the points along the tangential direction using the minimum bending energy criterion in TPSRELW v.149 [59]. For the partial Procrustes superimposition, each landmark configuration is first centered at the origin (0, 0) by subtracting the centroid from each (x, y) landmark coordinate for each configuration. The centroid size (the square root of the summed square distance of all landmarks from the centroid) is then scaled to 1 by dividing the centered coordinates by the original centroid size. A partial Procrustes superimposition does not allow the centroid size to vary from 1, unlike a full Procrustes superimposition, and is typically preferred [66], [67]. Specimens are then aligned to the consensus, or mean shape, calculated from a multiple iterative procedure by which all specimens are superimposed on one another. Specimens are then individually rotated to minimize the added squared differences of landmark coordinates between each specimen and the consensus, after which a new consensus is calculated. This procedure is iterated until the consensus stops changing significantly after multiple subsequent iterations [67]. Once specimens are aligned to the consensus shape, they are said to be in partial Procrustes superimposition.

The thin-plate spline function was then used to express shape differences of the specimens from the calculated consensus in terms of the bending energy matrix (see [63], [68]), the eigenvectors of which are called the principal warps [63]. The partial warp scores are calculated by projecting the Procrustes aligned landmark configurations onto the principal warps and are non-uniform shape changes that describe local variation in shape. A relative warp analysis (RWA) is a form of principal components analysis (PCA) using a variance-covariance matrix of the matrix of partial warp scores [69] and is commonly used in geometric morphometric studies (e.g., [26], [70]). We chose to run a PCA using the statistical software R [71] because it allows for optimization of graphical output, despite not being useful for statistical analyses of sliding, semi-landmarks [72]. Initial results showed that the RWA produced by TPS and the PCA produced from the principal warps in R of the extant data set were identical. Marine and winged taxa were excluded from the extinct analyses because their lifestyles differ from the terrestrial dinosaurs examined. While this initial broad taxonomic and ecological sample of extant taxa was unlikely to be useful for testing hypotheses about strictly terrestrial extinct taxa, it allowed us to test if the radial head morphology exhibited a significant phylogenetic signal (using Blomberg et al.’s [73] *K*) using a matrix of the relative warp scores for all extant taxa examined in the R package phytools [74]. The phylogeny and branch lengths were derived from published molecular phylogenies [75]–[96] (File S2).

Semilandmarks reduce the available degrees of freedom such that traditional methods for determining significant differences between groups are inappropriate because the number of free variables exceeds the number of degrees of freedom [72]. To determine significant differences between our groups, we used the IMP program TwoGroupMac7 [60] to perform a permutation test of the partial Procrustes distances using Goodall’s *F*-test. Permutation tests on the partial Procrustes distances also avoid assumption of normality of the landmarks [72]. The output of the PCA is, therefore, unrelated to our test for statistically significant differences, so similarities or differences in morphospace, which are affected by all specimens included, may or may not agree with statistical differences. To correct for multiple pair-wise comparisons, we used a Bonferroni correction to establish our criterion for a truly significant between-group difference, which is calculated by dividing 0.05 by the number of comparisons performed, resulting in significance levels of 0.002, 0.0018, and 0.0011 depending on the analysis.

## Results

### Extant Analyses

Cetaceans were significantly different in their angle of curvature from marsupials, eutherian mammals, squamates, and pinnipeds (Table 1). Birds and eutherian mammals also differed in angle of curvature (*p*<0.002), but no other differences among extant groups were found (Table 1). When chamaeleonids and non-chamaeleonid squamates were separated, a significant difference was also found between eutherians and non-chamaeleonid squamates (Table S2).

**Table 1:** Results from the Kruskal-Wallis test on extant taxa.

	Mo	Ma	Eu	S	Cr	Ce	Pi	A
Mo	–	–	–	–	–	–	–	–
Ma		–	–	–	–	–	–	–
Eu			–	–	–	–	–	–
S				–	–	–	–	–
Cr					–	–	–	–
Ce		*	*	*		–	–	–
Pi						*	–	–
A			*					–

Significant differences between extant taxa based on angle of curvature using a Bonferroni-corrected *p*-value. Blank spaces represent non-significant differences between groups.

* *p*<0.002; Mo = monotremes, Ma = marsupials, Eu = terrestrial eutherians, S = squamates, Cr = crocodylians, Ce = cetaceans, Pi = pinnipeds and sirenians, A = avians.

**Figure 4:**
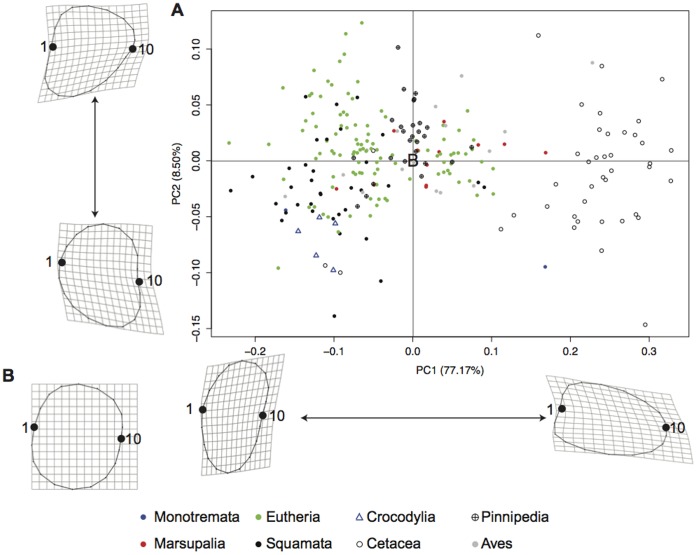
Extant radial head PCA. A graphical representation of the first two principal components from the analysis containing extant taxa (A). The shape at the origin is represented by the consensus (B). Shape change along the principal component axes is shown with the location of the consensus shown at the origin (B). Landmarks 1 (middle of the ulnar articular surface) and 10 are labeled.

When the shape of the radial head was examined, the first PC axis in the extant-only analysis summarized over 77% of the variance in the data set (Fig. 4A). The broken stick model showed that PC1 summarized significant variance and PC2 (8.59%) summarizes a proportion of variance slightly more than would be expected by chance alone. Along the positive PC1 axis, the radial head morphology becomes elongate relative to the ulna and stretches away from the articular surface (Fig. 4), and the radial head compresses toward the articular surface along the negative PC1 axis (Fig. 4). The positive PC2 axis represents somewhat triangular radial head morphology (Fig. 4), and the negative PC2 axis represents a more reniform morphology (Fig. 4).

In the bootstrap analyses of extant groups, many significant differences were recovered (Table 2, File S3). Cetaceans and eutherians were significantly different from all groups except monotremes (*p*<0.0018; Table 2). Marsupials and the pinniped+sirenian group were significantly different from all groups except Aves and monotremes (*p*<0.0018; Table 2). Crocodilians and squamates were significantly different from all other groups except monotremes and each other (*p*<0.0018; Table 2). When chalmaeleonids were separated from all other squamates, they were significantly different from cetaceans (*F = *53.08, *p*<0.0011) and non-chamaeleonid squamates (*F = *11.63, *p*<0.0011) (File S3). Non-chamaeleonid squamates were significantly different from marsupials (*F = *48.33, *p*<0.0011), eutherians (*F = *36.93, *p*<0.0011), cetaceans (*F = *367.7, *p*<0.0011), pinnipeds and sirenians (*F = *48.66, *p*<0.0011), and birds (*F = *33.8, *p*<0.0011) (File S3). Clemente et al. [97] found no correlation between posture and phylogeny among species of *Vanarus*, and we expected similar results here. A test of phylogenetic signal resulted in a significant *K*-value (*p*<0.01) for the relative warps scores of our extant data set. However, when mammals and reptiles were analyzed separately, non-significant differences were recovered (mammals, *K = *58.02, *p = *1; reptiles, *K = *16.09, *p* = 1), meaning that the phylogenetic signal is between, but not within, mammals and reptiles.

**Table 2:** Results from the Bootsrap analyses of extant taxa.

	Mo	Ma	Eu	S	Cr	Ce	Pi	A
Mo	–	–	–	–	–	–	–	–
Ma	3.08	–	–	–	–	–	–	–
Eu	4.63	10.63*	–	–	–	–	–	–
S	4.42	30.77*	28.31*	–	–	–	–	–
Cr	2.62	34.95*	13.65*	2.67	–	–	–	–
Ce	15.83	81.48*	362.83*	321.59*	101.19*	–	–	–
Pi	4.39	6.9*	9.25*	30.48*	26.79*	222.45*	–	–
A	1.77	1.73	10.28*	23.84*	14.03*	82.76*	3.66	–

F-scores and significant differences (*) between extant taxa based on radial head morphology based on the partial Procrustes distances in IMP with a Bonferroni-corrected *p*-value.

* *p*<0.00178; Mo = monotremes, Ma = marsupials, Eu = terrestrial eutherians, S = squamates, Cr = crocodylians, Ce = cetaceans, Pi = pinnipeds and sirenians, A = avians.

### Testing Forelimb Posture in Dinosaurs

The Kruskal-Wallis test with a Bonferroni-corrected *p*-value found a significant difference in the angle measurements between limited and active pronators (*p*<0.002). Active pronators were also significantly different from ceratopsians, ornithopods, and sauropodomorphs (Table S3). No other significant differences were found (Table 3). When ornithopods were separated, a Bonferroni-corrected significant difference (*p*<0.0018) was still recovered between hadrosaurids and active pronators, but non-hadrosaurid ornithopods were not significantly different from any group (Table 3), possibly due to small sample size (*n = *4). When chamaeleonids and therians were separated, therians were significantly different (*p*<0.0018) in their angle of curvature from limited pronators, ceratopsians, thyreophorans and sauropodomorphs, and chamaeleonids were significantly different from thyreophorans (Table S4).

**Table 3:** Results from the Kruskal-Wallis test with non-avian dinosaurs.

	Sp	P	C	NH	H	Thy	Sa	The
Sp	–	–	–	–	–	–	–	–
P	*	–	–	–	–	–	–	–
C		*	–	–	–	–	–	–
NH				–	–	–	–	–
H		*			–	–	–	–
Thy						–	–	–
Sa		*					–	–
The								–

Significant differences between sprawling taxa unable to rotate the radius about the ulna, parasagittal taxa able to rotate the radius about the ulna (to differing degrees), and extinct non-avian dinosaurs based on angle of curvature with a Bonferroni-corrected *p*-value. Blank spaces represent non-significant differences between groups.

* *p*<0.00178; Sp = sprawled, P = parasagittal, C = ceratopsian, NH = non-hadrosaurid ornithopod, H = hadrosaurid, Thy = thyreophoran, Sa = sauropodomorph, The = theropod.

**Figure 5:**
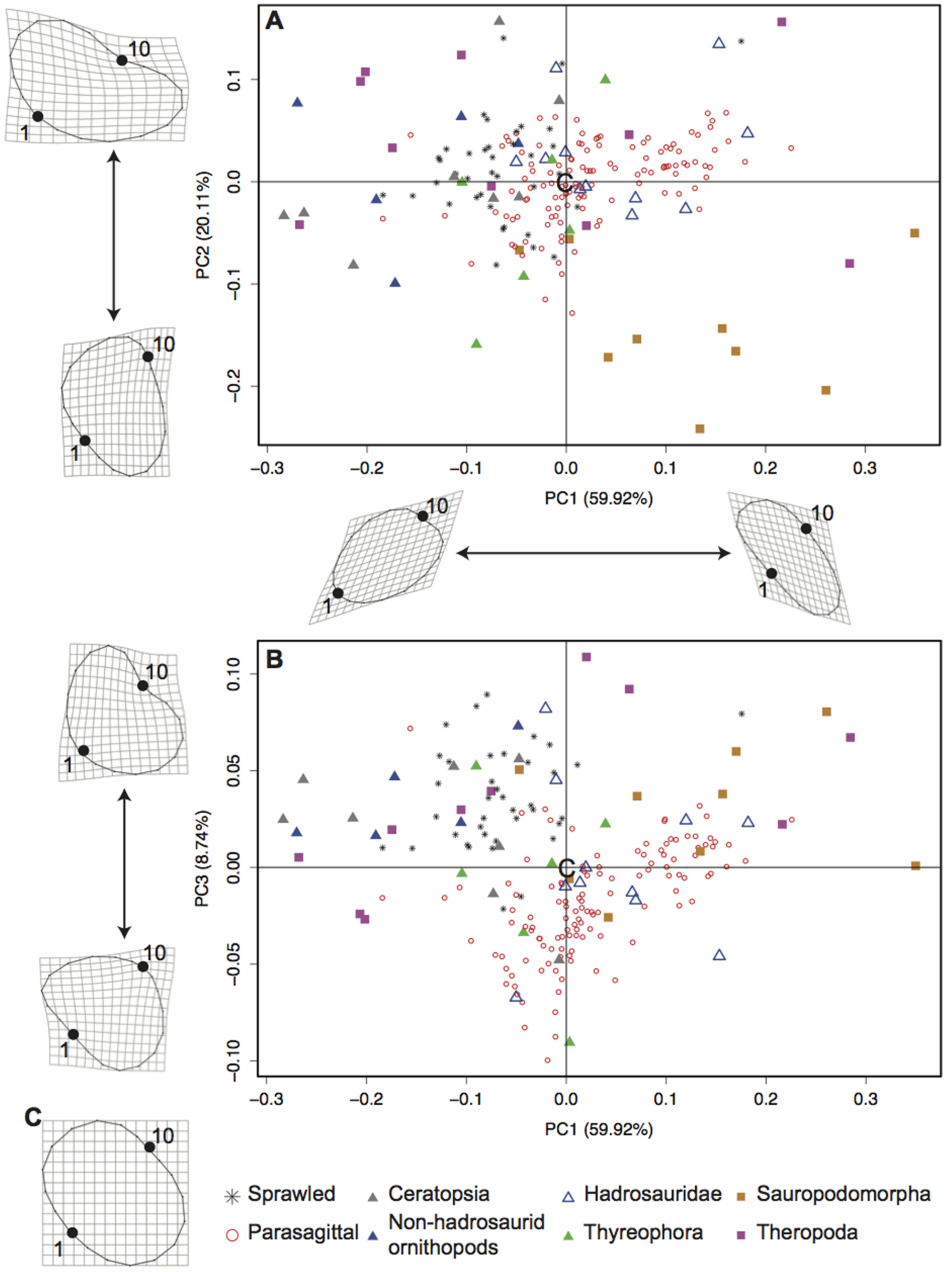
Terrestrial radial head PCA. Principal component scores from the analysis of terrestrial taxa. PC1 vs. PC2 (A) and PC1 vs. PC3 (B) have 95% convex hulls representing the sprawled and parasagittal taxa. The origin of each axis is represented by the shape of the consensus (C). Shape change along the principal component axes is shown with the location of the consensus shown at the origin (C). Landmarks 1 (middle of the ulnar articular surface) and 10 are labeled.

In the PCA of radial head morphology, PC1 summarized 59.78% of the variance (Fig. 5A). A scree plot revealed that the first three axes summarized more of the variation than should be expected by random chance alone. The positive PC1 axis again represents a radial head morphology elongated away from the ulna (Fig. 5), whereas the negative PC1 axis represents a morphology compressed toward the articular surface (Fig. 5). PC2 (20.13%) represents a reniform radial head morphology positively (Fig. 5) and a triangular morphology with a less curved articular surface negatively (Fig. 5). PC3 summarizes 8.80% of the variation (Fig. 5B) and represents a shape change in the radial head from a generally semi-circular radial head with a flat ulnar articular surface (negative, Fig. 5) to a curved ulnar articular surface with an indent cranial to the ulna (positive, Fig. 5). Together, the first three principal components summarize 88.71% of the variation in this analysis.

PC1 and PC3 best separate extant limited and active pronators (Fig. 5). The negative PC1 axis and positive PC2 axis are occupied by extant limited pronators, and the positive PC1 axis and negative PC3 axis are occupied by extant active pronators. Ceratopsians fall within the morphospace of limited pronators and farther negative than extant limited pronators on PC1. Thyreophorans inhabit a morphospace between the two mobility groups. Theropods and ornithopods do not obviously fall within one group or another, similar to the birds from the extant PCA. A breakdown of the ornithopods into non-hadrosaurid ornithopods and hadrosaurids showed that bipedal non-hadrosaurid ornithopods fall close to extant limited pronators, meaning their ability to actively pronate and supinate was limited, and hadrosaurids fall with extant active pronators.

Bootstrap analyses recovered significant differences (*p*<0.002) in the radial head morphology of extant limited and active pronators (Table 4, File S3). Extant active pronators were also significantly different from ceratopsians (*F = *27.8, *p*<0.0018), sauropodomorphs (*F = *42.02, *p*<0.0018), and theropods (*F = *11.69, *p*<0.0018), but not from ornithopods (*F = *5.78, *p = *0.0028) or thyreophorans (*F = *3.83, *p = *0.0224) (File S3). The only dinosaur group that was significantly different from extant limited pronators were sauropodomorphs (*F = *51.85, *p*<0.0018), which was significantly different from all other dinosaur groups (Table 4; File S3). However, when ornithopods were divided, hadrosaurids were significantly different from extant limited pronators (*F = *13.92, *p*<0.0018), ceratopsians (*F = *11.43, *p*<0.0018), and non-hadrosaurid ornithopods (*F = *13.95, *p*<0.0018), and non-hadrosaurid ornithopods were significantly different from extant active pronators (*F = *26.3, *p*<0.0018) (Table 4). Within the group of extant active pronators, eutherians and marsupials were found to be significantly different from each other (*F = *10.63, *p*<0.0018), despite our hypothesis that both clades should exhibit active pronation and supination capabilities (Table 2). However, when we tested each group individually against the dinosaur groups, similar results were obtained (File S3). When therians and chamaeleonids were separated, therians were significantly different from limited pronators (*F = *46.38, *p*<0.0018), ceratopsians (*F = *27.85, *p*<0.0018), sauropodomorphs (*F = *11.73, *p*<0.0018), and theropods (*F = *42.28, *p*<0.0018), but chamaeleonids were only different from limited pronators (*F = *8.12, *p*<0.0018) (Table S3).

**Table 4:** Results from the Bootsrap analyses of terrestrial extant taxa and non-avian dinosaurs.

	Sp	P	C	NH	H	Thy	Sa	The
Sp	–	–	–	–	–	–	–	–
P	46.14*	–	–	–	–	–	–	–
C	4.84	27.8*	–	–	–	–	–	–
NH	7.96	26.3*	0.74	–	–	–	–	–
H	13.92*	2.36	11.43*	13.95*	–	–	–	–
Thy	3.78	3.83	3.56	6.08	3.34	–	–	–
Sa	51.85*	42.02*	21.62*	17.9*	11.18*	9.03*	–	–
The	0.91	11.69*	0.86	1.37	3.21	1.17	11.51*	–

F-scores and significant differences (*) between terrestrial taxa based on radial head morphology based on the partial Procrustes distances in IMP with a Bonferroni-corrected *p*-value.

* *p*<0.00178; Sp = sprawled, P = parasagittal, C = ceratopsian, NH = non-hadrosaurid ornithopod, H = hadrosaurid, Thy = thyreophoran, Sa = sauropodomorph, The = theropod.

## Discussion

### The Radius as an Indicator of Posture and Forearm Rotational Mobility

The goal of this study was to quantitatively predict and constrain antebrachial posture and utility in extinct dinosaurs using a model composed of a phylogenetically broad sample of extant taxa. The limited number of significant differences for the angle of curvature in the extant data set may likely be due to low sample sizes for some groups, as a significant difference was found when taxa were grouped into mobility categories in the terrestrial analysis including extinct taxa. The PCA of extant taxa appears to be highly skewed by cetacean radii, where the shape along the positive PC1 axis (Fig. 4) is morphologically similar to that of a cetacean radius. While pinnipeds do not share this morphology, their radial head morphology lies between terrestrial taxa with active and restricted pronation, and this placement in morphospace reflects their limited ability to rotate the radius about the ulna (e.g., [98]). Marine mammals have a unique mode of life very different from terrestrial taxa (including dinosaurs) and would have been inappropriate to compare with obligatory terrestrial taxa. Marsupials, however, also appear to fall in a transitional morphospace between active and restricted pronators based on their radial head morphology, which suggests the amount of active pronation in this group is more limited than in eutherians. We grouped them with eutherian taxa, however, because they have previously been considered parasagittal and are likely capable of some degree of antebrachial rotation (see [99]).

The bootstrap analyses found multiple significant differences among extant groups (*p*<0.0018). The lack of significant differences between monotremes and other groups is likely due to the low number of species in Monotremata (*n = *2). The difference between the aquatic clades is unsurprising given that cetaceans are completely aquatic and pinnipeds are amphibious. Only two sirenians were used in our study, so their placement in either of these groups is unlikely to dramatically affect the results. The significant difference between marsupials and eutherians might have been driven by an over representation of macropods in the marsupial dataset (*n = *8 out of 12). Macropods do pronate their manus [100], so this result may simply reflect the amount of variation present in the eutherian dataset. This interpretation is supported by relatively similar results when the groups are compared to other groups in both analyses (Table 1, File S3). The lack of significant difference between squamates and crocodilians demonstrate that groups primarily composed of taxa with restricted active (mammalian-style) pronation do not significantly differ in their radial morphology, while the significant differences observed between ecologically or posturally dissimilar groups, such as squamates and cetaceans or squamates and eutherians, are expected given these difference. The significant difference in radial head morphology between chamaeleonid and non-chamaeleonid squamates further validates our results given the unique arboreal lifestyle of chamaeleonids [3], [42]. Therefore, our results do indicate that our metrics of radial morphology allow us to predict forearm posture and mobility in extant taxa.

### Forelimb Posture in Non-Avian Dinosaurs and the Evolution of Quadrupedality

Resolution of the forelimb posture debate in dinosaurs is important to test many hypotheses about the acquisition of large body size in all vertebrates. As mammals increase in size, their parasagittal limbs, those placed under the body near the midline, shift from a crouched posture to an upright posture to maintain safety factors [1], [2], [32], [101]. Safety factors represent the ratio of a stress at which a structure will fail and the actual stress it must endure (e.g., [102]). The higher the safety factor, the more tolerant an organism is to high, abnormal forces acting upon its limbs. This same trend toward a more upright limb posture with increased body size has been found in the hindlimbs of birds [103]. A parasagittal limb has many mechanical advantages, particularly in reducing the force muscles must exert and thus the force bones must resist (e.g., [1]). A parasagittal hindlimb is generally accepted in dinosaurs, and this posture agrees with what is expected for limbs of large-bodied animals [1], [2], [32]. However, if quadrupedal dinosaurs had a sprawling forelimb, as has been suggested [18], [20], [21], this arrangement would not only be novel among terrestrial vertebrates, but it would also challenge our understanding of limb bone loadings at large body masses [1], [2].

Bipedality is the ancestral state for all dinosaurian taxa (e.g., [104]). While we were mostly interested in the radial morphology of quadrupedal dinosaurs, non-avian theropods and non-iguanodontian ornithopods were included in our analyses as representatives of the ancestral radial morphology. Previous functional studies suggest some non-avian theropods may have had a limited ability to actively pronate their manus [105], [106]. Non-avian theropods show a high range of variation in the angle of curvature and spanned from the extreme negative to the extreme positive morphospace of PC1 (Fig. 5), suggesting greater differences forelimb function compared to that of quadrupedal dinosaurs [107]. This result is unsurprising given lack of locomotor constraints present on the forelimbs of bipedal taxa and suggests that a wider sampling of theropods could give greater insight to clade-specific differences in forelimb utility. However, their radial head morphology is still significantly different from that of active pronators (*p*<0.01), thereby supporting previous findings [105], [106]. The lower disparity in both angle of curvature and radial head morphology for non-hadrosaurid ornithopods is likely due to a small sample size, but significant differences between non-hadrosaurid ornithopods and parasagittal mammals in radial head morphology were still recovered (Table 4, File S3) suggesting that active pronation in non-iguanodontian ornithopod taxa examined here was unlikely.

Quadrupedal dinosaurs share a number of morphological convergences [12], [14], although their limbs functioned very differently among major clades [13]. We found that the angle of curvature of the radius in ornithopods, ceratopsians, and sauropodomorphs was more similar to that of extant taxa unable to cross their radii over their ulnae than to those that can actively rotate the radius about the ulna. Differences were also observed when the radial head morphology of ceratopsians, non-hadrosaurid ornithopods, and sauropodomorphs were compared to parasagittal taxa (*p*<0.0018), further supporting a reconstruction of a parallel radius and ulna. The radial head morphology of hadrosaurid ornithopods was found to be significantly different from sprawled taxa (*p*<0.0018), but without a significant difference in the angle of curvature as well, antebrachial rotation would not have been possible in this taxon. The difference between hadrosaurid and non-hadrosaurid ornithopods suggests a change in radial head morphology occurred during the acquisition of quadrupedality in this group, and we suggest that the forearms of ornithopods should be studied in more detail along this transition to determine the functional reason for this shape change.

Sauropodomorphs were significantly different from many other taxa in radial head morphology (*p*<0.002; Table 4, File S3), but this result is probably a product of our methodology. The midpoint of the radioulnar articulation would have been altered in sauropodomorphs by their developed craniolateral processes, which is more developed than that of other quadrupedal dinosaurs. The craniolateral process of the ulna, convergent in all quadrupedal dinosaur taxa, has been suggested to limit pronation and supination ability by cupping the radial head [12], [27]. However, our results indicate that active pronation would have been severely limited in some quadrupedal dinosaurs by radial morphology alone, suggesting the craniolateral process may have instead acted to stabilize the radius during locomotion.

Thyreophorans were not found to be significantly different from either extant group (parasagittal taxa or sprawling taxa) except when marsupials and eutherians were divided, in which case they were significantly different than marsupials in radial head morphology (*p = *0.0004; File S3). Rather than suggesting this pattern could be attributable to an ‘intermediate’ amount of active pronation and a radius that may slightly cross the ulna, it is likely the small thyreophoran sample size is hindering our ability to statistically conclude patterns of radial morphology. However, the angle of curvature in the thyreophoran sample is near 90 degrees (Table S1), and we predict that a significant difference between thyreophorans and parasagittal taxa would be found given a more comprehensive sample. It is also possible that these results indicate a varanid-like pronation style in thyreoporan locomotion, but further tests examining this hypothesis should be explored.

Using the radius alone to assess forelimb posture and utility has limitations. First, because no alternative exists, we are limited to the sprawled/parasagittal and presence/absence of active pronation ability dichotomies. Many authors have noted that problems lie with the current dichotomy of either sprawled or parasagittal forelimb posture (e.g., [16], [19]). Considering that there would have been a gradual shift from the sprawled posture seen in many sauropsids to a parasagittal posture seen in many mammals, this criticism is not surprising and can also be applied to the dichotomy for pronation ability. Some postural studies will use terms such as “semi-sprawled” or “semi-erect” (e.g., [20], [108]–[110]) but these terms are unspecific, theoretically including any posture in which the resting position of the humero-ulnar joint is between 90 and 180 degrees. While better classification that incorporates a continuum of postures is obviously needed (see [111]), none yet exists for use in the current study. There is also ambiguous terminology used when assigning active pronation and supination abilities in species (e.g., [112], [113]) and when discussing forelimb evolution (e.g., [43]). Due to a lack of methodology specifically designed to quantify this range, we are unable to correct for it here and thus classified antebrachial rotation and posture dichotomously.

The evolution of pronation ability in extant taxa may be causally linked to arboreality. Arboreal or scansorial locomotion has been hypothesized for the common ancestor of therian mammals, and this lifestyle is associated with increased pronation and supination ability [114]–[117]. While it is beyond the scope of this study to comment on the selective pressures acting on increased forearm rotation, the convergence of a semi-parasagittal gait in the chameleon step-cycle [3], [42] supports a functional connection between this lifestyle and forearm function. If this hypothesis were to be supported by future studies, it would be unclear how pronation/supination would have been selected for in dinosaurs, given that no quadrupedal dinosaur is hypothesized to have been arboreal.

The bipedal ancestry of dinosaurs may have instead allowed them to develop novel quadrupedal forelimb postures not seen in any extant, primary quadrupeds. Fujiwara [23] recognized that the forelimb of *Triceratops* may have been arranged so that the manus was directed laterally rather than cranially, but pronation of the manus was achieved by obtaining a ‘sauropod-like’ metacarpal configuration [27]. The laterally directed manus was also hypothesized by Rasmussen [118] for *Ouranosaurus* and by Senter [52] for hadrosaurids and has been supported by some ichnological studies [119] but not all [120]. Because of the direct link between the radius and manus, the orientation of the radius should directly affect the orientation of the manus. A ‘sauropod-like’ metacarpal configuration has also been found in thyreophorans [121], [122] and has been used to argue obligate quadrupedality in hadrosaurids [12]. An upright posture with a cranially-directed manus is only observed in taxa that cross their radii over their ulnae, or reduce the ulna to such a degree that they essentially have only one antebrachial element (i.e., ungulates). Therefore, we suggest that the convergent ‘sauropod-like’ manus structure seen in all quadrupedal dinosaurs could have functioned to direct the manus craniolaterally to partially pronate the manus without crossing the radius over the ulna, creating a novel, possibly more columnar forelimb posture than what is seen in antebrachium of most mammals. If supported by future research, this conclusion could give insight into the methodological limitations of using extant analogues (mammals and reptiles) when inferring the morphological adaptations for body size and locomotor habits in secondarily quadrupedal dinosaurian taxa.

## Acknowledgments

For access to specimens in museum collections, we thank C. Mehling (AMNH), M. Lamana and A. Henrici (CM), A. Shinya, W. Simpson, W. Stanley, A. Resetar, K. Kelly, and J. Holstein (FMNH), D. Evans, K. Seymour, and B. Iwama (ROM), B. Strilisky (RTMP), and C. Potter, A. Wynn, J. Jacobs, J. Ososky, L. Gordon, and C. Peterson (USNM). We thank K. Brink, C. Brown, N. Campione, and D. Larson for reviewing previous versions of this manuscript. We also thank J. White and H. Parks for their support during the original data collection and interpretation and J. Arbour, C. Brown, N. Campione, T. Cullen, D. Evans, D. Fraser, S. Maidment, A. Morhardt, H. Sheets, P. Polly, and A. Watanabe for helpful discussions. Finally, we thank Shin-ichi Fujiwara, Peter Dodson, and an anonymous reviewer for greatly improving the quality of this manuscript.

## Supporting Information

Table S1 **Dataset used for analyses.** Specimen numbers, breakdowns used for the analyses, angle measurements, and principal components scores for all specimens used. Numerical classifications for extant-only and terrestrial-only analyses align with group order in in-text tables.
(XLS)

Table S2 **Results from the Kruskal-Wallis test of extant taxa with chamaeleonids and non-chamaeleonid squamates separated.** Significant differences between sprawling taxa unable to rotate the radius about the ulna and parasagittal taxa, able to rotate the radius about the ulna with a Bonferroni-corrected *p*-value. Blank spaces represent non-significant differences between groups.
(DOCX)

Table S3 **Results from the Kruskal-Wallis test with non-avian dinosaurs (ornithopods grouped together).** Significant differences between sprawling taxa unable to rotate the radius about the ulna, parasagittal taxa able to rotate the radius about the ulna (to differing degrees), and extinct non-avian dinosaurs based on angle of curvature with a Bonferroni-corrected *p*-value. Blank spaces represent non-significant differences between groups.
(DOCX)

Table S4 **Results from the Kruskal-Wallis test with non-avian dinosaurs (chamaeleonids and therians split).** Significant differences between sprawling taxa unable to rotate the radius about the ulna, parasagittal taxa able to rotate the radius about the ulna (to differing degrees), and extinct non-avian dinosaurs based on angle of curvature with a Bonferroni-corrected *p*-value. Blank spaces represent non-significant differences between groups.
(DOCX)

File S1 **Landmark data for all specimens included in this study.**
(TPS)

File S2 **Composite phylogeny used for test of phylogenetic signal.**
(NEX)

File S3 **Results of additional bootstrap analyses discussed in text.** F-scores and significant differences (*) between terrestrial taxa based on radial head morphology based on the partial Procrustes distances in IMP with a Bonferroni-corrected *p*-value. Blank spaces represent comparisons that were made during previous analyses.
(XLSX)

## References

[1] Biewener AA (1989) Scaling body support in mammals: limb posture and muscle mechanics. Science 245. 10.1126/science.2740914 2740914

[2] Biewener AA (1990) Biomechanics of mammalian terrestrial locomotion. Science 250: 1097–1103. 2251499 10.1126/science.2251499

[3] Fischer MS, Krause C, Lilje KE (2010) Evolution of chameleon locomotion, or how to become arboreal as a reptile. Zoology 113: 67–74. 19747806 10.1016/j.zool.2009.07.001

[4] Shubin NH, Daeschler EB, Jenkins FA (2006) The pectoral fin of Tiktaalik roseae and the origin of the tetrapod limb. Nature 440: 764–771. 16598250 10.1038/nature04637

[5] Warren RD, Crompton RH (1997) Locomotor ecology of Lepilemur edwardsi and Avahi occidentalis. American Journal of Physical Anthropology 104: 471–486. 9453697 10.1002/(SICI)1096-8644(199712)104:4<471::AID-AJPA4>3.0.CO;2-V

[6] Higham TE, Davenport MS, Jayne BC (2001) Maneuvering in an arboreal habitat: the effects of turning angle on the locomotion of three sympatric ecomorphs of Anolis lizards. Journal of Experimental Biology 204: 4141–4155. 11809788 10.1242/jeb.204.23.4141

[7] Gatesy SM, Middleton KM (1997) Bipedalism, flight, and the evolution of theropod locomotor diversity. Journal of Vertebrate Paleontology 17: 308–329.

[8] Middleton KM, Gatesy SM (2000) Theropod forelimb design and evolution. Zoological Journal of the Linnean Society 128: 149–187.

[9] Thorpe SKS, Holder RL, Crompton RH (2007) Origin of human bipedalism as an adaptation for locomotion on flexible branches. Science 316: 1328–1331. 17540902 10.1126/science.1140799

[10] Schmitt D (2003) Insights into the evolution of human bipedalism from experimental studies of humans and other primates. Journal of Experimental Biology 206: 1437–1448. 12654883 10.1242/jeb.00279

[11] Tallman M (2012) Morphology of the distal radius in extant hominoids and fossil hominins: Implications for the evolution of bipedalism. The Anatomical Record 295: 454–464. 22262653 10.1002/ar.22405

[12] Maidment SCR, Linton DH, Upchurch P, Barrett PM (2012) Limb-bone scaling indicates diverse stance and gait in quadrupedal ornithischian dinosaurs. PLoS ONE 7: e36904. 22666333 10.1371/journal.pone.0036904 PMC3358279

[13] Maidment SCR, Barrett PM (2012) Does morphological convergence imply functional similarity? A test using the evolution of quadrupedalism in ornithischian dinosaurs. Proceedings of the Royal Society B: Biological Sciences 279: 3765–3771. 22719033 10.1098/rspb.2012.1040 PMC3415913

[14] Maidment SCR, Barrett PM (In press) Osteological correlates for quadrupedality in ornithischian dinosaurs. Acta Palaeontologica Polonica.

[15] Nesbitt SJ, Sidor CA, Irmis RB, Angielczyk KD, Smith RMH, et al (2010) Ecologically distinct dinosaurian sister group shows early diversification of Ornithodira. Nature 464: 95–98. 20203608 10.1038/nature08718

[16] Fujiwara S, Hutchinson JR (2012) Elbow joint adductor moment arm as an indicator of forelimb posture in extinct quadrupedal tetrapods. Proceedings of the Royal Society B: Biological Sciences 279: 2561–2570. 22357261 10.1098/rspb.2012.0190 PMC3350707

[17] Bakker RT (1986) The dinosaur heresies: new theories unlocking the mystery of the dinosaurs and their extinction. New York: Kensington Publishing Corp. 481 p.

[18] Johnson RE, Ostrom JH (1995) The forelimb of Torosaurus and an analysis of the posture and gait of ceratopsian dinosaurs. In: Thomason J, editor. Functional Morphology in Vertebrate Paleontology. Cambridge: Cambridge University Press. 205–218.

[19] Paul GS, Christiansen P (2000) Forelimb posture in neoceratopsian dinosaurs: implications for gait and locomotion. Paleobiology 26: 450–465.

[20] Dodson P, Farlow JO. The forelimb carriage of ceratopsid dinosaurs. In: Wolberg DL, Stump E, Rosenberg GD, editors; 1997; Academy of Natural Sciences, Philidephia. 393–398.

[21] Russell LS (1935) Musculature and functions in the Ceratopsia. Bulletin of the National Museum of Canada 77: 39–48.

[22] Thompson S, Holmes R (2007) Forelimb stance and step cycle in *Chasmosaurus irvinensis* (Dinosauria: Neoceratopsia). Palaeontologia Electronica 10: 17p.

[23] Fujiwara S (2009) A reevaluation of the manus structure in Triceratops (Ceratopsia: Ceratopsidae). Journal of Vertebrate Paleontology 29: 1136–1147.

[24] Bonnan MF, Senter P (2007) Were the basal sauropodomorph dinosaurs Plateosaurus and Massospondylus habitual quadrupeds? In: Barrett PM, Batten DJ, editors. Evolution and palaeobiology of early sauropodomorph dinosaurs: Special Papers in Palaeontology. 139–155.

[25] Bonnan MF, Yates AM (2007) A new description of the forelimb of the basal sauropodomorph Melanorosaurus: implications for the evolution of pronation, manus shape and quadrupedalism in sauropod dinosaurs. In: Barrett PM, Batten DJ, editors. Evolution and palaeobiology of early sauropodomorph dinosaurs: Special Papers in Palaeontology. 157–168.

[26] Bonnan MF (2007) Linear and geometric morphometric analysis of long bone scaling patterns in Jurassic Neosauropod dinosaurs: their functional and paleobiological implications. The Anatomical Record 290: 1089–1111. 17721981 10.1002/ar.20578

[27] Bonnan MF (2003) The evolution of manus shape in sauropod dinosaurs: implications for functional morphology, forelimb orientation, and phylogeny. Journal of Vertebrate Paleontology 23: 595–613.

[28] Yates AM, Bonnan MF, Neveling J, Chinsamy A, Blackbeard MG (2010) A new transitional sauropodomorph dinosaur from the Early Jurassic of South Africa and the evolution of sauropod feeding and quadrupedalism. Proceedings of the Royal Society B: Biological Sciences 277: 787–794. 19906674 10.1098/rspb.2009.1440 PMC2842739

[29] Reisz RR, Evans DC, Sues HD, Scott D (2010) Embryonic skeletal anatomy of the sauropodomorph dinosaur Massospondylus from the Lower Jurassic of South Africa. Journal of Vertebrate Paleontology 30: 1653–1665.

[30] Bonnan MF (2004) Morphometric analysis of humerus and femur shape in Morrison sauropods: implications for functional morphology and paleobiology. Paleobiology 30: 444–470.

[31] Molnar RE (1977) Analogies in the evolution of combat and display structures in ornithopods and ungulates. Evolutionary Theory 3: 165–190.

[32] Biewener AA (2005) Biomechanical consequences of scaling. Journal of Experimental Biology 208: 1665–1676. 15855398 10.1242/jeb.01520

[33] Senter P (2007) Analysis of forelimb function in basal ceratopsians. Journal of Zoology 273: 305–314.

[34] Bonnan MF, Sandrik JL, Nishiwaki T, Wilhite D, Elsey RM, et al (2010) Calcified cartilage shape in archosaur long bones reflects overlying joint shape in stress-bearing elements: Implications for nonavian dinosaur locomotion. The Anatomical Record 293: 2044–2055. 21046673 10.1002/ar.21266

[35] Holliday CM, Ridgely RC, Sedlmayr JC, Witmer LM (2010) Cartilaginous epiphyses in extant archosaurs and their implications for reconstructing limb function in dinosaurs. PLoS ONE 5: e13120. 20927347 10.1371/journal.pone.0013120 PMC2948032

[36] Fujiwara S, Taru H, Suzuki D (2010) Shape of articular surface of crocodilian (Archosauria) elbow joints and its relevance to sauropsids. Journal of Morphology 271: 883–896. 20544876 10.1002/jmor.10846

[37] Fujiwara S, Kuwazuru O, Inuzuka N, Yoshikawa N (2009) Relationship between scapular position and structural strength of rib cage in quadruped animals. Journal of Mammalogy 270: 1084–1094. 10.1002/jmor.10744 19378269

[38] Alexander RM (1982) Locomotion of animals. New York: Blackie Glasgow. 163 p.

[39] MacLeod N, Rose KD (1993) Inferring locomotor behavior in Paleogene mammals via eigenshape analysis. American Journal of Science 293: 300–355.

[40] Hildebrand M, Goslow GE (2001) Analysis of vertebrate structure. New York: Wiley. 635 p.

[41] Flower WH (1885) An Introduction to the Osteology of the Mammalia. London: Macmillan.

[42] Peterson JA (1984) The locomotion of Chamaeleo (Reptilia: Sauria) with particular reference to the forelimb. Journal of Zoology 202: 1–42.

[43] Polly PD (2007) Limbs in mammalian evolution. In: Hall BK, editor. Fins into Limbs: Evolution, Development and Transformation. Chicago: University of Chicago Press. 245–268.

[44] Iwaniuk AN, Pellis SM, Whishaw IQ (1999) The relationship between forelimb morphology and behaviour in North American carnivores (Carnivora). Canadian Journal of Zoology 77: 1064–1074.

[45] Andersson KI (2004) Elbow-joint morphology as a guide to forearm function and foraging behaviour in mammalian carnivores. Zoological Journal of the Linnean Society 142: 91–104.

[46] Iwaniuk AN, Whishaw IQ (1999) How skilled are the skilled limb movements of the raccoon (Procyon lotor)? Behavioural brain research 99: 35–44. 10512570 10.1016/s0166-4328(98)00067-9

[47] Figueirido B, Janis CM (2011) The predatory behaviour of the thylacine: Tasmanian tiger or marsupial wolf? Biology Letters 7: 937–940. 21543392 10.1098/rsbl.2011.0364 PMC3210661

[48] Galton PM (1970) The posture of hadrosaurian dinosaurs. Journal of Paleontology 44: 464–473.

[49] Landsmeer JMF (1983) The mechanism of forearm rotation in Varanus exanthematicus. Journal of Morphology 175: 119–130. 10.1002/jmor.1051750202 30068061

[50] Meers MB (2003) Crocodylian forelimb musculature and its relevance to Archosauria. The Anatomical Record Part A: Discoveries in Molecular, Cellular, and Evolutionary Biology 274: 891–916. 10.1002/ar.a.10097 12973714

[51] Nishi S (1916) Zur vergleichenden Anatomie der eigentlichen (genuinen) Rückenmuskeln. Gegenbaurs Morphol Jb 50: 167–318.

[52] Senter P (2012) Forearm orientation in Hadrosauridae (Dinosauria: Ornithopoda) and implications for museum mounts. Paleontologica Electronica 15: 10p.

[53] Horner JR, Weishampel DB, Forster CA (2004) Hadrosauridae. In: Weishampel DB, Dodson P, Osmolska H, editors. The Dinosauria (2nd ed). 2nd Ed. ed. Berkeley, CA: University of California Press. 438–463.

[54] Dilkes DW (2001) An ontogenetic perspective on locomotion in the Late Cretaceous dinosaur *Maiasaura peeblesorum* (Ornithischia: Hadrosauridae). Canadian Journal of Earth Sciences 38: 1205–1227.

[55] Brett-Surman MK, Wagner JR (2006) Discussion of character analysis of the appendicular anatomy in Campanian and Maastrichtian North American hadrosaurids-variation and ontogeny. In: Carpenter K, editor. Horns and Beaks: Ceratopsian and Ornithopod Dinosaurs. Bloomington, Indiana: Indiana University Press. 135–169.

[56] Taylor ME (1974) The functional anatomy of the forelimb of some African Viverridae (Carnivora). Journal of Morphology 143: 307–335. 4837745 10.1002/jmor.1051430305

[57] Abràmoff MD, Magalhães PJ, Ram SJ (2004) Image processing with ImageJ. Biophotonics international 11: 36–42.

[58] Siegel S, Castellan NJ (1988) Nonparametric Statistics for the Behavioral Sciences. New York: McGraw-Hill Book Company.

[59] Rohlf FJ (2006) TPS software series. Department of Ecology and Evolution, State University of New York, Stony Brook.

[60] Sheets HD, Zelditch ML, Swiderski D (2004) IMP - Intergrate Morphometric Package. 7 ed. Buffalo, N.Y.: Published by the Authors.

[61] Perez SI, Bernal V, Gonzalez PN (2006) Differences between sliding semi-landmark methods in geometric morphometrics, with an application to human craniofacial and dental variation. Journal of Anatomy 208: 769–784. 16761977 10.1111/j.1469-7580.2006.00576.x PMC2100233

[62] Bookstein FL (1996) Landmark methods for forms without landmarks: localizing group differences in outline shape. Medical Image Analysis 1: 225–243. 10.1016/s1361-8415(97)85012-8 9873908

[63] Bookstein FL (1997) Morphometric tools for landmark data: geometry and biology. New York: Cambridge Univ Press.

[64] Gower JC (1975) Generalized procrustes analysis. Psychometrika 40: 33–51.

[65] Rohlf FJ, Slice D (1990) Extensions of the Procrustes method for the optimal superimposition of landmarks. Systematic Biology 39: 40–59.

[66] Slice DE (2001) Landmark coordinates aligned by Procrustes analysis do not lie in Kendall’s shape space. Systematic Biology 50: 141–149. 12116591 10.1080/10635150119110

[67] Rohlf FJ (1999) Shape statistics: Procrustes superimpositions and tangent spaces. Journal of Classification 16: 197–223.

[68] Gunz P, Mitteroecker P, Bookstein F (2005) Semilandmarks in three dimensions. In: Slice DE, editor. Modern morphometrics in physical anthropology. New York: Kluwer Academic/Plenum Publishing. 73–98.

[69] Rohlf FJ (1993) Relative warp analysis and an example of its application to mosquito wings. In: Marcus LF, Bello E, García-Valdascases A, editors. Contributions to morphometrics. Madrid: CSIC. 131–159.

[70] Campione NE, Evans DC (2011) Cranial growth and variation in *Edmontosaurus* (Dinosauria: Hadrosauridae): implications for Latest Cretaceous megaherbivore diversity in North America. PLoS ONE 6: e25186. 21969872 10.1371/journal.pone.0025186 PMC3182183

[71] R-Development-Core-Team (2010) R: A Language and Environment for Statistical Computing. 2.12.0 ed. Vienna, Austria: R Foundation for Statistical Computing.

[72] Webster M, Sheets HD (2010) A practical introduction to landmark-based geometric morphometrics. In: Alroy J, Hunt G, editors. Quantitative Methods in Paleobiology Paleontological Society Papers. 163–188.

[73] Blomberg SP, Garland Jr T, Ives AR (2003) Testing for phylogenetic signal in comparative data: behavioral traits are more labile. Evolution 57: 717–745. 12778543 10.1111/j.0014-3820.2003.tb00285.x

[74] Revell LJ (2012) phytools: an R package for phylogenetic comparative biology (and other things). Methods in Ecology and Evolution 3: 217–223.

[75] Arnason U, Gullberg A, Janke A, Kullberg M (2007) Mitogenomic analyses of caniform relationships. Molecular Phylogenetics and Evolution 45: 863–874. 17919938 10.1016/j.ympev.2007.06.019

[76] Koepfli KP, Jenks SM, Eizirik E, Zahirpour T, Valkenburgh BV, et al (2006) Molecular systematics of the Hyaenidae: relationships of a relictual lineage resolved by a molecular supermatrix. Molecular Phylogenetics and Evolution 38: 603–620. 16503281 10.1016/j.ympev.2005.10.017

[77] Cione AL, Azpelicueta MM, Bond M, Carlini AA, Casciotta JR, et al.. (2000) Miocene vertebrates from Entre Ríos province, eastern Argentina. El Neógeno de Argentina 14.

[78] Amer SAM, Kumazawa Y (2005) Mitochondrial genome of Pogona vitticepes (Reptilia; Agamidae): control region duplication and the origin of Australasian agamids. Gene 346: 249–256. 15716001 10.1016/j.gene.2004.11.014

[79] Brown J, Rest J, García-Moreno J, Sorenson M, Mindell D (2008) Strong mitochondrial DNA support for a Cretaceous origin of modern avian lineages. BMC Biology 6: 6. 18226223 10.1186/1741-7007-6-6 PMC2267772

[80] Brown JW, Payne RB, Mindell DP (2007) Nuclear DNA does not reconcile ‘rocks’ and ‘clocks’ in Neoaves: a comment on Ericson et al. Biology Letters 3: 257–260. 17389215 10.1098/rsbl.2006.0611 PMC2464679

[81] Ericson PGP, Anderson CL, Britton T, Elzanowski A, Johansson US, et al (2006) Diversification of Neoaves: integration of molecular sequence data and fossils. Biology Letters 2: 543–547. 17148284 10.1098/rsbl.2006.0523 PMC1834003

[82] Fabre P, Rodrigues A, Douzery EJP (2009) Patterns of macroevolution among Primates inferred from a supermatrix of mitochondrial and nuclear DNA. Molecular Phylogenetics and Evolution 53: 808–825. 19682589 10.1016/j.ympev.2009.08.004

[83] Frostick LE, Reid I (1986) Evolution and sedimentary character of lake deltas fed by ephemeral rivers in the Turkana basin, northern Kenya. Geological Society, London, Special Publications 25: 113–125.

[84] Gonzalez J, Düttmann H, Wink M (2009) Phylogenetic relationships based on two mitochondrial genes and hybridization patterns in Anatidae. Journal of Zoology 279: 310–318.

[85] Johnson WE, Eizirik E, Pecon-Slattery J, Murphy WJ, Antunes A, et al (2006) The late Miocene radiation of modern Felidae: a genetic assessment. Science 311: 73–77. 16400146 10.1126/science.1122277

[86] Marshall LG, Sempere T (1991) The Eocene to Pleistocene vertebrates of Bolivia and their stratigraphic context: a review. Fósiles y facies de Bolivia 1: 631–652.

[87] Okajima Y, Kumazawa Y (2010) Mitochondrial genomes of acrodont lizards: timing of gene rearrangements and phylogenetic and biogeographic implications. BMC Evolutionary Biology 10: 141. 20465814 10.1186/1471-2148-10-141 PMC2889956

[88] Pereira SL, Baker AJ (2006) A molecular timescale for galliform birds accounting for uncertainty in time estimates and heterogeneity of rates of DNA substitutions across lineages and sites. Molecular Phylogenetics and Evolution 38: 499–509. 16112881 10.1016/j.ympev.2005.07.007

[89] Pyron RA (2010) A likelihood method for assessing molecular divergence time estimates and the placement of fossil calibrations. Systematic Biology 59: 185–194. 20525629 10.1093/sysbio/syp090

[90] Roos J, Aggarwal RK, Janke A (2007) Extended mitogenomic phylogenetic analyses yield new insight into crocodylian evolution and their survival of the Cretaceous-Tertiary boundary. Molecular Phylogenetics and Evolution 45: 663–673. 17719245 10.1016/j.ympev.2007.06.018

[91] Schulte II JA, Melville J, Larson A (2003) Molecular phylogenetic evidence for ancient divergence of lizard taxa on either side of Wallace’s Line. Proceedings of the Royal Society of London Series B: Biological Sciences 270: 597–603. 12769459 10.1098/rspb.2002.2272 PMC1691285

[92] Wiens JJ, Brandley MC, Reeder TW (2006) Why does a trait evolve multiple times within a clade? Repeated evolution of snakeline body form in squamate reptiles. Evolution 60: 123–141. 16568638

[93] Zarza E, Reynoso VH, Emerson BC (2008) Diversification in the northern neotropics: mitochondrial and nuclear DNA phylogeography of the iguana Ctenosaura pectinata and related species. Molecular ecology 17: 3259–3275. 18564087 10.1111/j.1365-294X.2008.03826.x

[94] Zrzavy J, Ricankova V (2004) Phylogeny of recent Canidae (Mammalia, Carnivora): relative reliability and utility of morphological and molecular datasets. Zoologica Scripta 33: 311–333.

[95] Fyler CA, Reeder TW, Berta A, Antonelis G, Aguilar A, et al (2005) Historical biogeography and phylogeny of monachine seals (Pinnipedia: Phocidae) based on mitochondrial and nuclear DNA data. Journal of Biogeography 32: 1267–1279.

[96] Bininda-Emonds OR, Cardillo M, Jones KE, MacPhee RDE, Beck RMD, et al (2007) The delayed rise of present-day mammals. Nature 446: 507–512. 17392779 10.1038/nature05634

[97] Clemente CJ, Withers PC, Thompson G, Lloyd D (2011) Evolution of limb bone loading and body size in varanid lizards. The Journal of Experimental Biology 214: 3013–3020. 21865513 10.1242/jeb.059345

[98] English AWM (1977) Structural correlates of forelimb function in fur seals and sea lions. Journal of Morphology 151: 325–352. 845968 10.1002/jmor.1051510303

[99] Sereno PC (2006) Shoulder girdle and forelimb in multituberculates: evolution of parasagittal forelimb posture in mammals. In: Carrano M, editor. Amniote Paleobiology: Perspectives on the Evolution of Mammals, Birds, and Reptiles: a Volume Honoring James Allen Hopson. Chicago: University of Chicago Press. 315–366.

[100] Harvey KJ, Warburton N (2010) Forelimb musculature of kangaroos with particular emphasis on the tammar wallaby Macropus eugenii (Desmarest, 1817). Australian Mammalogy 32: 1–9.

[101] Bertram JEA, Biewener AA (1990) Differential scaling of the long bones in the terrestrial Carnivora and other mammals. Journal of Morphology 204: 157–169. 2348461 10.1002/jmor.1052040205

[102] Beer FP, Johnston E (1981) Mechanics of Materials. New York: McGraw-Hill.

[103] Gatesy SM, Biewener AA (1991) Bipedal locomotion: effects of speed, size and limb posture in birds and humans. Journal of Zoology 224: 127–147.

[104] Benton MJ (2005) Vertebrate Palaeontology, 3rd ed. MaldenMA: Blackwell Publishing Company. 455 p.

[105] Senter P, Robins JH (2005) Range of motion in the forelimb of the theropod dinosaur Acrocanthosaurus atokensis, and implications for predatory behaviour. Journal of Zoology 266: 307–318.

[106] Carpenter K (2002) Forelimb biomechanics of nonavian theropod dinosaurs in predation. Palaeobiodiversity and Palaeoenvironments 82: 59–75.

[107] Middleton KM, Gatesy SM (2008) Theropod forelimb design and evolution. Zoological Journal of the Linnean Society 128: 149–187.

[108] Gambaryan PP, Kielan-Jaworowska Z (1997) Sprawling versus parasagittal stance in multituberculate mammals. Acta Palaeontologica Polonica 42: 13–44.

[109] Parchman AJ, Reilly SM, Biknevicius AR (2003) Whole-body mechanics and gaits in the gray short-tailed opossum Monodelphis domestica: integrating patterns of locomotion in a semi-erect mammal. Journal of Experimental Biology 206: 1379–1388. 12624172 10.1242/jeb.00267

[110] Reilly SM, Elias JA (1998) Locomotion in Alligator mississippiensis: kinematic effects of speed and posture and their relevance to the sprawling-to-erect paradigm. Journal of Experimental Biology 201: 2559–2574. 9716509 10.1242/jeb.201.18.2559

[111] Carrano MT (1999) What, if anything, is a cursor? Categories versus continua for determining locomotor habit in mammals and dinosaurs. Journal of Zoology 247: 29–42.

[112] Thewissen JGM, Hussain ST (2007) Postcranial Osteology of the most Primitive Artiodactyl: Diacodexis pakistanensis (Dichobunidae). Anatomia, Histologia, Embryologia 19: 37–48. 10.1111/j.1439-0264.1990.tb00876.x 2375509

[113] Argot C (2004) Evolution of South American mammalian predators (Borhyaenoidea): anatomical and palaeobiological implications. Zoological Journal of the Linnean Society 140: 487–521.

[114] Haines RW (1958) Arboreal or terrestrial ancestry of placental mammals. Quarterly Review of Biology 33: 1–23. 13542756 10.1086/402206

[115] O’Leary MA, Bloch JI, Flynn JJ, Gaudin TJ, Giallombardo A, et al (2013) The Placental Mammal Ancestor and the Post–K-Pg Radiation of Placentals. Science 339: 662–667. 23393258 10.1126/science.1229237

[116] Huxley TH (1880) Arboreal ancestry of the marsupials. Proceedings of the Zoological Society of London: 655–668.

[117] Matthew WD (1904) The arboreal ancestry of the Mammalia. The American Naturalist 38: 811–818.

[118] Rasmussen ME (1998) Notes on the morphology and the orientation of the forelimb of Ouranosaurus nigeriensis. Oryctos 1: 127–130.

[119] Wright JL (1999) Ichnological evidence for the use of the forelimb in iguanodontid locomotion. Special Papers in Palaeontology 60: 209–219.

[120] Lockley MG, Hunt AP (1999) Dinosaur tracks: And other fossil footprints of the western United States: Columbia University Press.

[121] Senter P (2010) Evidence for a sauropod-like metacarpal configuration in stegosaurian dinosaurs. Acta Palaeontologica Polonica 55: 427–432.

[122] Senter PJ (2010) Evidence for a sauropod-like metacarpal configuration in ankylosaurian dinosaurs. Acta Palaeontologica Polonica 55: 427–432.

