# Supplementary material for: Forearm Posture and Mobility in Quadrupedal Dinosaurs
Source: PLoS One. 2013 Sep 18;8(9):e74842. doi: 10.1371/journal.pone.0074842 (PMC3776758; doi:10.1371/journal.pone.0074842)
Supplement: Table S2 — Results from the Kruskal-Wallis test of extant taxa with chamaeleonids and non-chamaeleonid squamates separated. Significant differences between sprawling taxa unable to rotate the radius about the ulna and parasagittal taxa, able to rotate the radius about the ulna with a Bonferroni-corrected p-value. Blank spaces represent non-significant differences between groups. (DOCX) [file pone.0074842.s002.docx]

|  | Mo | Ma | Eu | S | Ch | Cr | Ce | Pi |
| --- | --- | --- | --- | --- | --- | --- | --- | --- |
| Mo | - | - | - | - | - | - | - | - |
| Ma |  | - | - | - | - | - | - | - |
| Eu |  |  | - | - | - | - | - | - |
| S |  |  | * | - | - | - | - | - |
| Ch |  |  |  |  | - | - | - | - |
| Cr |  |  |  |  |  | - | - | - |
| Ce |  | * | * | * | * |  | - | - |
| Pi |  |  |  |  |  |  | * | - |

* *p* < 0.0018; Mo = monotremes, Ma = marsupials, Eu = eutherians, S = non-chamaeleonid squamates, Ch = chameleonids, Cr = crocodylians, Ce = cetaceans, Pi = pinnipeds and sirenians
