# Supplementary material for: Forearm Posture and Mobility in Quadrupedal Dinosaurs
Source: PLoS One. 2013 Sep 18;8(9):e74842. doi: 10.1371/journal.pone.0074842 (PMC3776758; doi:10.1371/journal.pone.0074842)
Supplement: Table S3 — Results from the Kruskal-Wallis test with non-avian dinosaurs (ornithopods grouped together). Significant differences between sprawling taxa unable to rotate the radius about the ulna, parasagittal taxa able to rotate the radius about the ulna (to differing degrees), and extinct non-avian dinosaurs based on angle of curvature with a Bonferroni-corrected p-value. Blank spaces represent non-significant differences between groups. (DOCX) [file pone.0074842.s003.docx]

|  | Sp | Eu | C | O | Thy | Sa | The |
| --- | --- | --- | --- | --- | --- | --- | --- |
| Sp | - | - | - | - | - | - | - |
| Eu | * | - | - | - | - | - | - |
| C |  | * | - | - | - | - | - |
| O |  | * |  | - | - | - | - |
| Thy |  |  |  |  | - | - | - |
| Sa |  | * |  |  |  | - | - |
| The |  |  |  |  |  |  | - |

* *p* < 0.002; Sp = sprawled, Eu = terrestrial eutherian, C = ceratopsian, O = ornithopod, Thy = thyreophoran, Sa = sauropodomorph, The = theropod
